# Ongoing and Novel Challenges in Kidney Transplantation: Therapeutic Approaches to Non-Immunological Risk Factors for Allograft Loss

**DOI:** 10.3390/life16020248

**Published:** 2026-02-02

**Authors:** Michele Provenzano, Roberta Arena, Ida Gagliardi, Lilio Hu, Chiara Ruotolo, Gemma Patella, Giuseppe Pezzi, Rosita Greco, Valeria Grandinetti, Rocco Malivindi, Michele Di Dio, Olga Baraldi, Giorgia Comai, Luca De Nicola

**Affiliations:** 1 Nephrology, Dialysis and Renal Transplant Unit, Department of Pharmacy, Health and Nutritional Sciences, University of Calabria, Rende-Hospital ‘SS. Annunziata’, 87100 Cosenza, Italy; roberta.arena@aspcs.it (R.A.); ida.gagliardi@aspcs.it (I.G.); gemma.patella@aspcs.it (G.P.); r.greco@aocs.it (R.G.); rocco.malivindi@unical.it (R.M.); michele.didio@unical.it (M.D.D.); 2 Department of Medical and Surgical Sciences (DIMEC), Alma Mater Studiorum University of Bologna, 40138 Bologna, Italy; olga.baraldi3@unibo.it (O.B.); giorgia.comai2@unibo.it (G.C.); 3 Nephrology and Dialysis Unit, Ospedale Santa Maria delle Croci, AUSL Romagna, viale Randi 5, 48121 Ravenna, Italy; 4 Renal Unit, DAMSS, University of Campania “Luigi Vanvitelli”, 80138 Naples, Italy; chiara.ruotolo@unicampania.it (C.R.); luca.denicola@unicampania.it (L.D.N.); 5 Department of Medical and Surgical Sciences, Magna Graecia University of Catanzaro, 88100 Catanzaro, Italy; giuseppe.pezzi@unicz.it; 6 Nephrology, Dialysis and Kidney Transplant Unit, IRCCS Azienda Ospedaliero-Universitaria di Bologna, 40138 Bologna, Italy; valeria.grandinetti@aosp.bo.it

**Keywords:** kidney transplantation, allograft loss, anemia, hypertension, diabetes, proteinuria

## Abstract

In recent decades, the rate of kidney transplantation has risen significantly, leading to better outcomes in terms of cardiovascular and overall mortality for patients with kidney failure. Although kidney transplantation represents the most effective therapeutic option, it is not devoid of the risk of failure. Immunological and non-immunological risk factors are involved. These factors often interact and may act synergistically, ultimately influencing graft longevity and patient survival. Both contribute to long-term transplant outcomes; however, non-immunological factors, representing a significant clinical challenge, will be the focus of our review. Of the numerous non-immunological risk factors, for clarity and to avoid overextending the discussion, only those most closely associated with chronic kidney disease have been considered: hypertension, anemia, diabetes mellitus, proteinuria, electrolyte and acid–base imbalances, and impaired bone mineralization. Hypertension is reported in approximately 90% of kidney transplant recipients, often related to immunosuppressive therapy and residual renal dysfunction, and it is strongly associated with reduced graft survival. Anemia affects approximately 20–51% of these patients, contributing to cardiovascular morbidity and a more rapid decline in graft function, as does pre-existing diabetes mellitus. Proteinuria has a prevalence ranging from 7.5% to 45%, depending on the established target, and is a significant negative prognostic factor. Metabolic complications are also frequent; for example, hyperkalemia has an incidence of 25–44%, and metabolic acidosis has a prevalence of 12–58%. In our review, each of these factors is analyzed in terms of clinical impact, etiopathogenic mechanism, and available therapeutic management.

## 1. Introduction

Kidney transplantation (KT) represents the best therapeutic option for patients with kidney failure (KF), and it is associated with better outcomes in terms of overall survival in comparison with hemodialysis or peritoneal dialysis [1]. International guidelines emphasize the importance of timely assessment of kidney transplant eligibility, framing this process as an integral part of an optimal care pathway aimed at improving the outcomes of patients with chronic kidney disease [2]. However, some pathologic conditions can occur even after kidney transplantation and influence the prognosis of KT patients. In addition to the well-known immunological factors (acute and chronic rejection, immunosuppressive drugs, etc.), non-immunological ones are also associated with all-cause mortality, decline in glomerular filtration, and graft failure. Among the non-immunological factors, topics such as obesity and cardiovascular disease would merit more extensive dedicated treatment in a separate manuscript. Instead, the factors that most commonly determine the progression of chronic kidney disease (CKD) were examined: anemia, hypertension (HT), proteinuria, mineral bone disease (MBD), and electrolyte and acid–base disturbances. These are all significant players in the management of acute and chronic kidney disease. However, their role and prognostic weight in the context of graft function preservation remain overlooked.

The literature selection included studies published between 2000 and 2025 for each topic area considered. Literature reviews, randomized controlled trials, and observational studies were included, with particular attention paid to the most recent clinical guidelines. To ensure more comprehensive and contextualized coverage of the topics, the analysis was extended, when necessary, to publications prior to this period.

## 2. Non-Immunological Risk Factors: Epidemiology and Clinical Dimension

### 2.1. Anemia

Anemia in kidney transplant recipients (KTRs), referred to as post-transplantation anemia (PTA), is defined according to the World Health Organization (WHO) criteria and the American Society of Transplantation as hemoglobin (Hb) levels less than 12 g/dL in women and less than 13 g/dL in men [3,4]. PTA is a common condition with a prevalence of approximately 20–51%. It is typically classified as “early” or “late” PTA based on the time of onset. Early PTA develops within 6 months after transplantation, while late PTA develops after 6 months [5]. The prevalence of early PTA is about 50%, while the prevalence of late PTA decreases to around 23–35% at various time points up to 8 years after transplantation [6].

Iron deficiency, caused by several factors, is recognized as the major contributor to early PTA. The causes of iron deficiency include depletion of iron stores before transplantation; perioperative blood loss; malnutrition; increased iron demand due to elevated erythropoietin (EPO) production by the allograft; resumption of the menstrual cycle; and the use of certain medications, such as anticoagulants or proton pump inhibitors, which can decrease iron absorption [5].

Late PTA, instead, is mainly related to the reduction in allograft function over time, secondary to a relatively lower production of EPO. However, other contributing factors may include iron deficiency or other nutritional deficiencies (such as folic acid and vitamin B12); low-grade inflammatory status; infections; immunosuppressive drugs like mycophenolate mofetil/mycophenolic acid, azathioprine, and mammalian target of rapamycin inhibitors (which promote hepcidin upregulation); medications affecting the renin–angiotensin–aldosterone system (RAAS), such as angiotensin-converting enzyme inhibitors (ACEIs) and angiotensin receptor blockers (ARBs) as angiotensin II enhances EPO secretion through tubule-interstitial ischemia; antimicrobial agents (trimethoprim-sulfamethoxazole) and antiviral agents (ganciclovir); donor age; and female sex [7].

Consequentially, PTA results in factors that compromise quality of life (i.e., reduced exercise capacity, weakness, chronic fatigue, and cognitive decline) and influence the risk of comorbidities and mortality. The reduced amount of oxygen delivered to tissues is the major cause of all the complications associated with anemia [8]. In KTRs, PTA is associated with several poor long-term clinical outcomes, such as estimated glomerular filtration rate (eGFR) decline, graft failure (GF), cardiovascular morbidity, and cardiovascular and all-cause mortality [9].

#### 2.1.1. Glomerular Filtration Rate Decline

Post-transplant anemia is associated with kidney function decline over time. Reduced oxygen-carrying capacity of blood due to anemia contributes to chronic hypoxia of kidney graft, which induces tubulointerstitial damage in an organ that may already be vulnerable due to ischemia–reperfusion injury [10,11]. In a retrospective cohort study of Gafter-Gvili and colleagues, 266 KTRs were followed for two years after transplantation. In anemic patients, eGFR declined by 2.26 mL/min/1.73 m^2^ after two years, while, in non-anemic patients, eGFR rose by 3 mL/min/1.73 m^2^, with a significant difference of 5.3 mL/min/1.73 m^2^ between the two groups [9]. In the prospective randomized trial CAPRIT (Correction of Anemia and Progression of Renal Insufficiency in Transplant Patients), the achievement of 13–15 g/dL of Hb reduced the decline rate of eGFR and creatinine clearance and the progression to KF in kidney recipients, suggesting an association between the GFR slope and Hb levels in these patients [12], as also highlighted in the prospective study of the group of Tsujita [13].

#### 2.1.2. Graft Failure

Anemia causes chronic inflammatory status (through activation of inflammatory cytokines IL-6 and TNF-α), which contributes to graft endothelial dysfunction and tubulointerstitial fibrosis. Moreover, oxidative stress due to hypoxia worsens glomerulosclerosis, accelerating graft failure [14]. Several trials documented the increased risk of graft failure in KTRs with PTA. A systematic review and meta-analysis of 11 observational studies including a total of 11,632 KTRs demonstrated that PTA significantly increases the risk of death-censored graft loss (DCGL), shown by the association between PTA and higher rates of graft loss and the link between the decrease in hemoglobin level and worse graft survival outcomes [15]. Furthermore, in a cohort of 1139 KTRs followed from 2002 to 2016 in the Rabin Medical Center, PTA at any time point was associated with the composite endpoint of mortality and graft failure [16], especially during the first three-year period. This association seems closely related to the severity of anemia. Analogous results also emerged from Austrian, Slovakian, and Japanese studies [17].

#### 2.1.3. Cardiovascular Morbidity and Mortality

Chronic anemia is responsible for the increase in the cardiac output (through risen preload, reduced afterload due to the falling of vascular resistances, positive inotropic and chronotropic effects, and an hyperdynamic state), leading to left ventricular eccentric hypertrophy. The augmented cardiac output also contributes to the arterial remodeling of central elastic arteries [18,19]. Left ventricular hypertrophy (LVH) is a recognized risk factor for cardiovascular disease (CVD), as well as cardiovascular and all-cause mortality. In a Canadian retrospective cohort study of 473 KTRs, anemia was identified as an independent risk factor for LVH one to five years after transplantation, as demonstrated by the inverse association between Hb levels and E/e’ (an echocardiography index of left ventricular filling pressure) [20].

In another trial conducted on 638 KTRs followed for up to 7 years, a lower Hb value was associated with a greater incidence of congestive heart failure and cardiovascular disease [21]. In contrast, the systematic review and meta-analysis by Mekraksakit and colleagues found an increased risk of Major Adverse Cardiovascular Events (MACEs) and cardiovascular death in KTRs with PTA compared to those without anemia [22].

Nevertheless, the evidence on the association between PTA and all-cause mortality remains conflicting [23,24]. While several studies have documented a link between increased all-cause mortality and anemia in KTRs, others did not find any significant association, probably due to methodological differences among the studies. Therefore, further research is needed on this topic [15,24,25].

#### 2.1.4. Management of Post-Transplant Anemia

Post-transplant anemia represents a modifiable risk factor in KTRs [26]. Its management starts with diagnosis and treatment of all reversible causes. The current guidelines do not define a specific hemoglobin target but suggest to maintain hemoglobin generally in the range of about 10–12 g/dL, avoiding routine targets ≥ 13 g/dL because of potential cardiovascular risks [27].

Regarding the treatment of iron deficiency anemia, whether intravenous (IV) or oral supplementation is better remains unclear. Oral iron supplementation is more convenient for its easier administration and its low cost despite the gastrointestinal side effects and the reduced intestinal absorption. However, IV iron is more effective and rapid in correcting iron deficiency but poses the risk of severe infusion reactions [28].

The use of erythropoiesis-stimulating agents (ESAs), especially in early PTA, should be assessed on a case-by-case basis and started only after iron stores have been restored and other underlying causes of anemia have been addressed. This is particularly important considering the high risk of malignancy in KTRs. Hypoxia-inducible factor prolyl hydroxylase (HIF-PH) inhibitors have recently become available for use in KTRs. They stimulate EPO production, reduce hepcidin levels, and improve iron homeostasis. However, clinical use in KTRs is limited [26].

### 2.2. Hypertension

**Figure 1:**
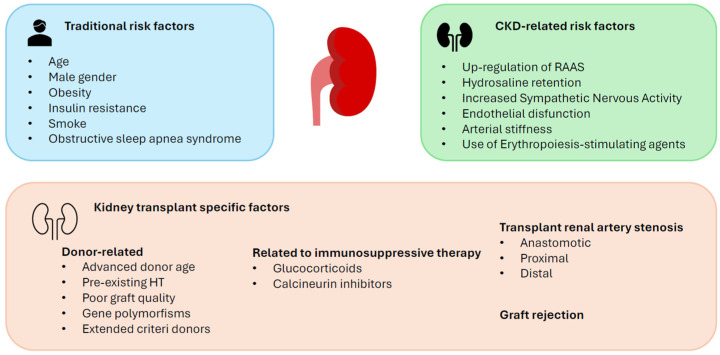
Determinants of hypertension after kidney transplantation. Schematic representation of the traditional cardiovascular risk factors, chronic kidney disease-related mechanisms, and transplant-specific determinants involved in the development of hypertension after kidney transplantation. RAAS, renin–angiotensin–aldosterone system; HT, hypertension; CKD, chronic kidney disease.

As a high-risk population, KTRs are addressed in the Kidney Disease Improving Global Outcomes (KDIGO) guidelines, which suggest maintaining systolic blood pressure (BP) < 130 mmHg and diastolic < 80 mmHg, and <90th percentile for gender, age, and height for adolescents and children [29]. Hypertension is defined as elevated BP values or normotension despite the use of antihypertensive drugs [30]. With a prevalence of about 90%, HT is very common in KTRs, and it is associated with a higher risk of GF, cardiovascular morbidity, and mortality [31]. Indeed, one of the leading causes of long-term morbidity and mortality in KTRs is CVD, with HT being a major contributing risk factor [31]. Uncontrolled BP is, in fact, associated with congestive heart failure, coronary heart disease, stroke, increased cardiovascular mortality, and poor allograft outcomes [31]. Unfortunately, the management of HT in KTRs remains an area that is poorly explored so far. This likely reflects the difficulties in both recognizing and effectively treating HT in KTRs [31]. Furthermore, abnormal blood pressure profiles, such as masked or white-coat, nocturnal, non-dipping, or isolated systolic/diastolic HT, are very common in these patients, suggesting that office BP measurements are less sensitive than 24 h ambulatory BP monitoring. The current evidence indicates that 24 h monitoring is more appropriate and sensitive for these patients, providing more information than office measurements alone [29,31]. Regarding the pathogenesis of HT, it can be broadly divided into traditional risk factors (normally present in the general population); risk factors associated with impaired kidney function (as observed in native CKD); and, finally, risk factors specific to KTRs (Figure 1) [31].

#### 2.2.1. Traditional Risk Factors

Hypertension in KTRs shares the same risk factors as in the general population, which include age, male gender, augmented body mass index (BMI), insulin resistance, obstructive sleep apnea, and a smoking habit. Many of these risk factors tend to worsen after transplantation, further increasing the risk of HT onset or worsening. Pre-existing HT before kidney transplantation has been recognized as a major contributing cause [32].

#### 2.2.2. CKD-Related Risk Factors

As renal function declines, we assist with an upregulation of the RAAS system with consequent systemic vasoconstriction (mediated by the direct vasoconstrictor angiotensin II) and hydro-saline retention, which results in extra-cellular fluid (ECF) expansion. These factors are associated with the onset of HT. Similarly, Sympathetic Nervous Activity (SNA) is augmented from the early stages of CKD and continues to rise as renal function declines, promoting increased peripheral vascular tone.

In the short-term post-transplantation, increased SNA is sustained by the native kidneys as long as the transplanted kidney remains denervated. However, over time, the transplanted kidneys undergo reinnervation, further contributing to increased SNA. Other CKD-related risk factors include endothelial dysfunction (reduced nitric oxide production and increased endothelin levels), arterial stiffness, and the administration of ESAs used to treat post-transplant anemia [32,33,34].

#### 2.2.3. Kidney Transplant-Specific Risk Factors

##### Donor-Related Factors

Several donor-related risk factors affect the onset of HT, including advanced age, pre-existing HT, obesity, or simply poor graft quality. Additionally, significant anthropometric differences between donors and recipients are relevant, often leading to glomerular hyperfiltration, increased intraglomerular pressure, and hypertrophy, all responsible factors for post-transplant HT and the decline in graft function [31].

Furthermore, some gene polymorphisms, like those coding for ABCC2, ABCB1, CYP3A5, and APOL-1, have been associated with an early graft dysfunction and an early onset of post-transplant HT [35,36].

In recent years, the introduction of transplantation from deceased donors with extended criteria (donor age > 60 years, or 50–59 years in the presence of at least two risk factors such as hypertension, cardiovascular death, or serum creatinine > 1.5 mg/dL) has highlighted a strong association between this donor category and reduced graft function, with a greater prevalence of post-transplant HT [37].

##### Factors Related to Immunosuppressive Therapy

The factors related to immunosuppressive therapy leading to HT include hydro-saline retention, increased systemic vascular resistances, arrhythmias, and atherosclerosis dependent on the use of high doses of glucocorticoids [32,38,39,40]; hydro-saline sodium retention and direct vascular effects of calcineurin inhibitors, supported by excess production of vasoconstrictors (i.e., thromboxane or endothelin-1) and reduced release of prostaglandins, with subsequent kidney hypoperfusion and stimulation of the RAAS [41,42]. However, emerging evidence is highlighting a lower incidence of adverse vascular effects and thus of post-transplantation HT with tacrolimus rather than with cyclosporin [32,42]. Moreover, both glucocorticoids and calcineurin inhibitors are associated with new onset of diabetes after transplantation.

##### Transplant Renal Artery Stenosis (TRAS)

A refractory HT associated with progressive graft dysfunction, or a sudden worsening of graft function after the administration of RAAS inhibitors, suggests renal artery stenosis. This is a very common complication, and its incidence is estimated at about 1–23% [43]. This complication can occur at any time after transplantation [44]. However, we can distinguish an early form that typically appears during the first months after surgery and is usually caused by mechanical traumas, and a late form, which seems to be associated with the evolution of an underlying donor atherosclerotic pathology. Other contributing factors to TRAS include comorbidities, dialysis duration before transplantation, donor type, acute rejection, delayed graft function, cold ischemic time, and peak systolic velocity.

Kidney transplant recipients with diabetes and hypertension are at higher risk as diabetes induces vascular damage through oxidative stress, chronic inflammation, endothelial dysfunction, atherosclerosis, and amplified immune activity, worsened by hypertension.

Longer dialysis duration contributes via chronic vascular stress, repeated vascular access, inflammation, and endothelial dysfunction. Transplants from deceased donors, especially with aortic patches, carry higher TRAS risk due to more complex surgery and prolonged ischemia.

Acute rejection triggers a strong immune response, causing endothelial injury and vascular damage. Delayed graft function similarly promotes inflammation and endothelial susceptibility to stenosis. Ischemia–reperfusion injury damages vascular structures directly.

Prolonged cold ischemia exacerbates cellular injury. Reperfusion induces oxidative stress and inflammatory activation.

Overall, multiple surgical, immunologic, and patient-related factors converge to increase TRAS risk [44]. Other contributing factors, although less frequently observed, include immunological and infectious factors (i.e., class II donor-specific antibodies and cytomegalovirus infection), which are responsible for immune-mediated endothelial injury.

TRAS can be classified on the basis of the anatomic site in anastomotic, proximal, and distal artery stenosis [31]. Regarding etiology, anastomotic arterial stenosis has been associated with surgically mediated damage; proximal stenosis seems to have a close correlation with atherosclerotic pathology of the donor, while, for the distal forms, the etiology is not yet completely clear.

Angiography is considered the diagnostic gold standard, but non-invasive imaging such as color Doppler ultrasonography and magnetic resonance angiography (MRA) can also be used to detect stenosis [44].

Management of TRAS consists of either endovascular or surgical procedures. Percutaneous transluminal balloon renal angioplasty (PTRA) is the most widely used procedure due to its reduced invasiveness and lower risk of complications. PTRA and stenting appears to be the most effective option. When a percutaneous procedure fails, or in the presence of anastomotic or severe stenosis, surgery becomes the preferred option. Several surgery techniques are available, ranging from the revision of the anastomosis to saphenous vein bypass grafting [45,46].

##### Antibody-Mediated Rejection (ABMR)

All types of rejection are associated with allograft dysfunction and subsequent HT [31]. However, rejection mediated by Angiotensin II Type 1-Receptor Activating (ATIIR) antibodies is characteristically associated with new-onset HT, worsening of post-transplant hypertension, and malignant HT [47].

##### Primary Hyperaldosteronism

Many studies suggest that the prevalence of primary hyperaldosteronism is about 5–20% in patients with hypertension [48,49]. Several case reports have documented the onset of primary hyperaldosteronism following kidney transplantation; however, there are no data about its prevalence and pathophysiology. These reports consistently document that hypokalemia accompanied by treatment-resistant HT in KTRs should raise suspicion of primary hyperaldosteronism [50].

##### Sodium Intake

Sodium intake is a novel and potentially modifiable risk factor for chronic kidney disease (CKAD) and cardiovascular events, although it is not yet sufficiently studied in these patients. High sodium intake is associated with several well-documented adverse effects, particularly on blood pressure, caused by mechanisms such as hypervolemia, increased sympathetic tone, oxidative stress, inflammation, endothelial dysfunction, vascular stiffness, and fibrosis. Consequently, a reduced sodium intake (<2.3 g/day) is recommended in patients with CKAD to achieve optimal blood pressure control [51].

Tacrolimus, a mainstay of maintenance immunosuppressive therapy, has been shown to induce salt-sensitive hypertension through upregulation of the phosphorylated sodium chloride cotransporter (NCC), mediated by with-no-lysine kinases (WNKs) [52]. Therefore, excessive sodium intake in these patients may contribute to common proteinuria or albuminuria, even independently of blood pressure levels, through mechanisms yet to be defined [53].

High sodium intake may also contribute to graft dysfunction through an induced pro-inflammatory state, oxidative stress, endothelial dysfunction, altered mitochondrial activity, and intestinal dysbiosis [51,54]. A high-salt diet can increase tissue sodium concentrations, influencing immune responses in local microenvironments and impairing the proliferation, differentiation, and activation of innate and adaptive immune cells [55,56].

The two main goals of antihypertensive therapy after kidney transplantation are preservation of graft function (or slowing the progression of renal disease) and reduction in cardiovascular disease risk [57]. Lifestyle modifications are generally recommended as first-line therapy, although there are only limited data on the outcomes of this intervention in KTR. To date, no randomized controlled clinical trial (RCT) has established the optimal antihypertensive regimen specifically for KTR. Therefore, the selection of antihypertensive therapy should be individualized, taking into account individual comorbidities and risk profiles [58]. 

Treatment

Calcium channel blockers (CCBs) reduce renal vasculature resistances, balancing the vasoconstrictive effects induced by calcineurin inhibitors (CNIs). Patients on CCBs present better kidney function and blood pressure control compared to those on other antihypertensive drugs, and CCBs are generally well tolerated. Although some studies have shown no superiority of CCBs over beta blockers and similar efficacy to alpha blockers and ACEIs, a systematic review of 17 different studies, involving overall 1255 KTRs, indicates that CCBs improve serum creatinine, eGFR, and may reduce the risk of graft failure by about 25% [59]. Despite this, the current guidelines do not recommend any specific class of antihypertensive as first-line therapy [29]. In head-to-head studies, CCBs significantly improved GFR by about 12 mL/min compared to ACEIs, with better outcomes [60].

In the early post-transplant period, drugs that inhibit RAAS are usually not administrated because ACEIs or ARBs can cause or worsen graft dysfunction due to their known hemodynamic effects. More critically, they may mask early signs of rejection and can lead to hyperkaliemia and even to anemia. However, their role in reducing proteinuria both in diabetic and non-diabetic patients and in slowing CKD progression is well established. RAAS inhibition in KTRs, however, remains a subject of debate. No significant differences were found in graft and patient survival, except in cases of severe proteinuria [60]. Therefore, the use of these drugs may be justified in patients with cardiovascular indications or in the presence of severe proteinuria, provided there are no contraindications [61].

Beta blockers, although not having significant effects on kidney function, are widely used in KTRs primarily to reduce CVD risk, as demonstrated by observational studies showing a significant survival benefit [62].

Generally, diuretics are not used as first-line therapy because of the risk of volume depletion, electrolyte imbalances, and subsequent potential renal injury, in particular for loop diuretics. Thiazide and thiazide-like diuretics can be effective medications, but they may also cause adverse metabolic effects, such as hyponatremia, hypokalemia, hypercalcemia, and hyperuricemia. Aldosterone antagonists can be considered, especially in KTRs with proteinuria. They have been shown to reduce proteinuria and slow the decline in GFR. However, it is important to keep in mind the increased risk of hyperkalemia [58].

To achieve the best possible control, some emerging agents can also be considered, such as the nonsteroidal mineralocorticoid receptor antagonist (nsMRA) finerenone or sodium–glucose cotransporter 2 inhibitors (SGLT-2i). These agents have demonstrated both nephroprotective and cardioprotective effects in patients with CKD, with and without diabetes [63,64,65]. In particular, SGLT-2 inhibitors have been shown to reduce blood pressure by an average of 3–5 mmHg in diabetics and 1–3 mmHg in non-diabetic patients. However, the current data are limited to small short-term studies, and more experience is needed with these new drugs [32].

### 2.3. Diabetes Mellitus

The prevalence of diabetes mellitus (DM) in the general population has increased significantly worldwide, having more than doubled compared to previous decades [66]. Diabetes is the leading cause of End-Stage Kidney Disease (ESKD). Patients with diabetes and CKD have increased morbidity and mortality due to CVD [67].

Kidney transplantation is the best therapeutic option, offering improved survival and better quality of life compared to dialysis treatment. However, in patients with pre-existing diabetes, it may lead to worsening glycemic control due to the immunosuppressive agents that are commonly used, such as CNIs, mammalian target of rapamycin inhibitors (mTORis), and corticosteroids, since they increase the phenomenon of insulin resistance and compromise insulin secretion [68].

The diabetogenic potential of CNIs has been shown to be dose-dependent, and tacrolimus is considered to be more diabetogenic than cyclosporine A (CsA). In fact, some patients without pre-existing diabetes may develop Post-Transplant Diabetes Mellitus (PDTM) [68].

Other post-transplant risk factors that may contribute to the onset of diabetes include infections such as hepatitis C, hypomagnesemia, resolution of uremic anorexia, and increased body weight [69].

Defined as diabetes diagnosed after kidney transplantation in patients without known pre-transplant diabetes, the incidence of PTDM can reach up to 83.7% within the first year after transplantation [70]. The diagnostic criteria for PTDM are the same as those used for diabetes mellitus in the general population. PTDM can be diagnosed when at least one of the following criteria is met: fasting plasma glucose ≥ 126 mg/dL (7.0 mmol/L); random plasma glucose ≥ 200 mg/dL (11.1 mmol/L) in the presence of classic symptoms of hyperglycemia; plasma glucose ≥ 200 mg/dL (11.1 mmol/L) two hours after a 75 g oral glucose tolerance test (OGTT); or glycated hemoglobin (HbA1c) ≥ 6.5% (48 mmol/mol). PTDM should not be diagnosed in the very early post-transplant period as perioperative hyperglycemia and the initial effects of immunosuppressive therapy may cause transient hyperglycemia. Diagnosis is preferably made once the patient is clinically stable and on established maintenance immunosuppression (typically ≥6–8 weeks or later) to avoid misdiagnosis in the early post-transplant phase.

However, HbA1c may be less reliable in the early post-transplant period due to anemia, altered red blood cell turnover, and the effects of immunosuppressive therapy; therefore, it should not be used as the sole diagnostic criterion during this phase [71].

In addition to the already known micro- and macrovascular complications related to suboptimal glycemic control, pre-existing diabetes appears to have a more severe impact on KT outcomes—such as cardiovascular disease, graft failure, and mortality—compared to post-transplant diabetes [72].

In addition, this is associated with an increased risk of post-transplant infections, delayed graft function (DGF), and early onset of rejection [68].

Lim and colleagues, in a cohort of 5248 KTRs, found that patients with pre-transplant diabetes had a higher incidence of MACEs and mortality compared to those who developed PDTM. The incidence of MACEs was up to three times higher in the pre-transplant diabetic group. While the incidence of cardiac and all-cause death remained constant for up to three years post-transplant, it increased considerably thereafter [72].

Similar findings were reported by the Australia and New Zealand Dialysis and Transplant Registry (ANZDATA) [73], which showed that KTRs with pre-transplant type 2 diabetes had worse cardiovascular and renal outcomes compared to those with PDTM. However, maintaining good glycemic control has been shown to preserve graft function and prevent the development and progression of vascular complications [73,74].

Despite the high prevalence of this patient population, optimal management remains unclear. Treatment can be complicated by the presence of severe autonomic neuropathy and other complications related to the long-standing disease. However, evidence suggests avoiding intensive therapies and target HbA1c values < 6.0% [29,75]. In the absence of large randomized trials, the choice is often based on expert opinions and recommendations extrapolated from RCTs of non-transplanted diabetic patients.

The choice of hypoglycemic agents is individualized considering the possible interactions with immunosuppressive drugs and the GFR impacting the metabolism and clearance of the drugs. The strategies to be adopted should aim to improve the functionality of pancreatic beta-cells and reduce insulin resistance [69]. The KDIGO guidelines set a target HbA1c of 7.0–7.5% [29]. Promoting a healthy lifestyle plays a key role in diabetic patients, but this has not yet been confirmed in KTRs. In the post-surgical period, insulin is the drug of choice since it protects β-cell function from hyperglycemia. Reduction in immunosuppressants is an option that cannot be considered in the first year due to the high risk of rejection [69].

#### Antidiabetic Drugs

- Metformin:

Metformin is generally considered to be the first-line treatment for diabetic patients but has a major limitation due to the risk of lactic acidosis when GFR declines, although it can be prescribed at adjusted dosages down to a GFR of 30 mL/min. The positive aspect is that metformin does not have interactions with immunosuppressive drugs, although it may potentiate the gastroenterological side effects of mycophenolate mofetil (MMF). If metformin is chosen, close monitoring of renal function over time is mandatory to minimize the risk of lactic acidosis [69].

• Sulfonylureas and Glinides:

Sulfonylureas and glinides carry the risk of accumulation with reduced renal function and have a higher risk of hypoglycemia compared to other antidiabetic agents.

• Glucagon-Like Peptide 1 Receptor Agonists (GLP1-RAs):

They promote glucose-dependent insulin secretion, inhibit glucagon release, suppress the sense of hunger and pancreatic β-cell apoptosis, and delay gastric emptying. Their renal clearance is not significant, making them generally safe to use in patients with reduced kidney function. Liraglutide, semaglutide, and dulaglutide have demonstrated significant benefits on renal and cardiovascular outcomes independent of their hypoglycemic effects. Although data in the transplant population remain limited, these drugs may exacerbate the gastrointestinal adverse effects of MMF [69].

• Dipeptidyl Peptidase 4 (DPP-4) Inhibitors:

They inhibit the enzyme that inactivates glucose-dependent insulinotropic polypeptide (GIP) and GLP-1. Linagliptin, sitagliptin, and vildagliptin have been used in KTRs with good results; however, they can have interactions with CNIs and have significant renal clearance.

• Sodium–Glucose Cotransporter 2 Inhibitors (SGLT-2i):

By inhibiting the sodium–glucose cotransporter at the proximal tubule, these drugs promote significant urinary glucose excretion with better glycemic control, fluids, body weight, and blood pressure. However, genitourinary tract infections and ketoacidosis are present as adverse events. These drugs have also demonstrated beneficial effects on cardiovascular and renal outcomes independent of glucose control [76,77]. Currently, the available data in renal transplantation are limited. However, they should be used with caution due to the increased risk of serious infections, such as Fournier’s gangrene, euglycemic acidosis, and hemodynamic-related Acute Kidney Injury (AKI), due to the reduction in blood volume supported by the diuretic effect in the presence of vasoconstriction of the afferent arteriole.

• Insulin Therapy:

Insulin remains the cornerstone in patients with type 1 DM and in cases where other hypoglycemic agents become insufficient. Technological progress has allowed this therapy to be optimized with the help of continuous glucose monitoring (CGM) systems and insulin pumps based on the parameters recorded by the CGM. This has allowed therapy to be personalized as much as possible and to obtain important results on outcomes, such as a lower incidence of hypoglycemia, better quality of life, and a reduction in mortality.

### 2.4. Proteinuria

Proteinuria is a common finding after kidney transplantation, with a prevalence ranging from 7.5% to 45% [78]. This large percentage variability depends on the threshold value used to define it [79]. In fact, the prevalence of proteinuria varies between 31 and 45%, using a threshold at the upper limits of the norm [80], drops to 19%, defining proteinuria as urinary excretion of proteins > 1 g/day [81], and it further reduces, to 13%, when the reference limit exceeds 2 to 3 g/day [82]. Although the threshold level considerably modifies the prevalence, there is no univocal definition of proteinuria in the renal transplant population. Therefore, it is appropriate to use the same values established for the general population [83]. Certainly, based on the many studies carried out, it can be stated with certainty that the prevalence of proteinuria is higher in transplant recipients than in the general population and that it is close to the level of the pre-dialysis population [84,85]. Post-transplant proteinuria in kidney recipients is influenced by donor and recipient factors, acute rejection, and immunosuppressive drugs, particularly mTOR inhibitors [78,79]. Furthermore, it can originate from both the native and the transplanted kidney [83], and the differential diagnosis can be supported by the extent of proteinuria, allowing early recognition of allograft pathologies that could compromise its survival [80,82]. In the following sections, the main causes of proteinuria after kidney transplantation will be addressed, distinguishing between proteinuria originating from the native or transplanted kidney as well as the prognostic value and the clinical–therapeutic management of proteinuria in transplant recipients.

#### 2.4.1. Risk Factors for the Development of Proteinuria in Kidney Transplant Recipients

**Figure 2:**
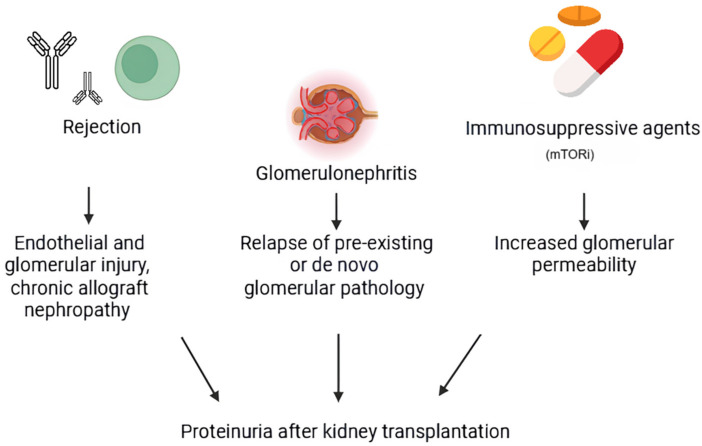
Pathophysiological mechanisms of proteinuria after kidney transplantation. Schematic representation of the main mechanisms contributing to proteinuria after kidney transplantation, including graft rejection-related injury, recurrence or de novo glomerulonephritis, and immunosuppressive therapy-associated increases in glomerular permeability. mTORi, mammalian target of rapamycin inhibitor.

Many factors predispose to the development of post-transplant proteinuria (Figure 2): a female donor, a male recipient, acute rejection, advanced age of the donor, cardiovascular death of the donor, and proteinuria in the donor [78,81]. Immunosuppressive agents have also been evaluated as potential factors for the development of proteinuria in the transplant recipient. Among all, sirolimus (SRL) was the first to be identified as a potentially reversible cause of proteinuria [79,86].

The proteinuria values associated with this immunosuppressive drug are generally <500 mg/day [80], and the studies carried out have not yet detected any allograft-specific pathologies associated with it [87].

Biopsy studies in proteinuric patients treated with SRL have highlighted the presence of glomerular diseases (focal segmental glomerulosclerosis, minimal-change glomerulonephritis, mesangial IgA glomerulonephritis, membranous glomerulonephritis, and membranoproliferative glomerulonephritis), as well as worsening of pre-existing glomerular pathology [83,87,88].

However, although proteinuria has been associated predominantly with SRL therapy, similar effects and similar levels of proteinuria have also been reported with everolimus [89,90].

Therefore, proteinuria could be generically defined as related to the use of mTOR inhibitors (mTORis) [83,91]. Specifically, proteinuria occurs in patients converted from a CNI to mTORi or in those with pre-existing proteinuria but not in patients with de novo use of mTORis [87,89,90]. For this reason, conversion to this class of drugs is not recommended in patients with pre-existing proteinuria > 800 mg/day [92].

#### 2.4.2. Proteinuria in Kidney Transplant Recipients: Does It Originate from the Native Kidney or the Transplanted Kidney?

The first issue to address in the case of post-transplant proteinuria is the origin [87]. Patients who have undergone pre-emptive transplantation or immediately after the start of dialysis therapy often have residual diuresis. If the original nephropathy was associated with proteinuria, in the earliest post-transplant phases, it is possible that the urine contains significant amounts of protein. Therefore, it becomes important to establish whether the proteinuria originates from the native kidney or the transplanted kidney [83].

Myslak M et al. provided guidelines for determining the source of post-transplant proteinuria. In the case of a functioning graft, proteinuria values > 3000 mg/day 3 weeks after transplantation are not attributable to the native kidneys, even in the case of pre-existing proteinuria; proteinuria > 1500 mg/day 1 year after transplantation and/or an increase in proteinuria > 500 mg/day within 1 year after transplantation is attributable to the transplanted kidney [93].

Generally, proteinuria originating from native kidneys resolves within the first 30 days post-transplant, although the mechanism responsible for this change is unknown [79]; persistent or worsening proteinuria is usually indicative of allograft pathology [93].

Biopsy studies on proteinuric transplant patients have shown that the most frequent histological alterations are glomerular disease, interstitial fibrosis, tubular atrophy, and acute rejection. Specifically, interstitial fibrosis and tubular atrophy are more common, with a mean prevalence of 31.6% compared to 16.8% for glomerulopathy [82,83,94]. Definitely, proteinuria values can guide the histological diagnosis [79]. In fact, higher proteinuria values (2716 ± 2889 mg/day) are typically associated with glomerulopathy, while patients with acute rejection or interstitial fibrosis present lower proteinuria values, generally <500 mg/day [80,82].

#### 2.4.3. The Prognostic Value of Proteinuria in Transplant Patients

Proteinuria is an independent risk factor for reduced graft survival and greater decline in kidney function but also for increased recipient mortality [95,96]. Transplant patients with proteinuria have a survival rate of 69%, compared to 93% for patients without proteinuria, as well as an increased risk of major cardiovascular (CV) events (myocardial infarction, stroke, and peripheral vascular disease) [83,97,98]. The cardiovascular risk increases independently of proteinuria levels [99].

Amer et al., in an important study, concluded that proteinuria was associated with reduced graft survival independent of the causative histologic alteration and glomerular filtration rate [80].

If high levels of proteinuria have been associated with worse prognosis, even low levels of proteinuria have been indicated as a negative prognostic factor and deserve attention [79]. In fact, in transplant recipients, proteinuria in the nephrotic range is associated with a significantly increased risk of transplant failure (>19 times), but also proteinuria values < 500 mg/day are correlated with a higher risk (>4 times) [87]. Furthermore, for every 1.0 g/day increase in proteinuria, there is a 16% increase in the risk of death from any cause [100].

For these reasons, the KDIGO 2009 guidelines suggest measuring proteinuria at least once within the first month after transplantation, then every 3 months in the first year, and annually thereafter [29]. Since the KDIGO guidelines do not provide recommendations on the best detection method, spot urine measurements could be valid screening tests, being simple and convenient for the patient and consistent with data from 24 h urine collections [29,101]. However, it is advisable to perform a 24 h urine collection in the case of a positive screening test [87].

In addition, all patients with new-onset proteinuria, persistent proteinuria, or increasing proteinuria after transplantation should undergo renal biopsy. In fact, some histological alterations can receive targeted therapies and can provide more detailed prognostic information [29,87]. Again, there are conflicting opinions on the definition of proteinuria. The KDIGO guidelines recommend biopsy for proteinuria > 3 g/day [29]; other sources lower the threshold to 1.5 g/day [87].

#### 2.4.4. Clinical–Therapeutic Management of Transplant Patients with Proteinuria

Evidence-based therapies are lacking for transplant patients [83]. Some sources suggest discontinuing mTOR inhibitors in patients with proteinuria > 500 mg/day and glomerular disease or with proteinuria < 500 mg/day that increases during follow-up [87]. Observational studies of RAAS blockade have produced mixed results [102]. Although it can be assumed that RAAS inhibitors have the same beneficial effect on transplant recipients as on non-transplant patients, published data demonstrate that this hypothesis cannot yet be confirmed. In fact, if on the one hand it has been shown that ACE inhibitors and sartans determine a significant reduction in proteinuria, on the other hand, a significant decrease in renal function has emerged, as well as a reduction in hemoglobin values up to levels associated with a risk > 24% of congestive heart failure and >16% of death [6,24,33,34]. For all these reasons, RAAS inhibitors are not currently recommended in these patients with the aim of improving their prognosis; further studies are needed [79]. Randomized trials have evaluated protein restriction as a therapeutic strategy for proteinuria [83]. Although albuminuria and proteinuria were significantly reduced during low-protein diets, the effect on long-term renal function or allograft survival has not been evaluated [79]. Therefore, protein restriction is currently not recommended in renal transplant patients with proteinuria, and its role remains to be defined [79,83].

### 2.5. Electrolyte and Acid–Base Disturbances

Renal transplantation corrects most of the electrolyte and acid–base abnormalities typical of advanced renal failure. However, in the post-transplant period, characteristic biochemical changes occur, different from those typical of CKD and secondary to multiple factors, including allograft function, immunosuppressive regimens adopted, and characteristic metabolic changes [103].

The most common electrolyte and acid–base disturbances are hyperkalemia, hypomagnesemia, hypercalcemia, hypophosphatemia, and metabolic acidosis [103].

Hypercalcemia and hypophosphatemia will be discussed in more detail in the section on bone mineral disease after transplantation. Below, hyperkalemia, acid–base disturbances, and hypomagnesemia will be discussed in more detail, analyzing their prevalence, pathophysiological mechanisms, the role of immunosuppressive drugs, clinical and prognostic implications, as well as monitoring and therapeutic management strategies.

#### 2.5.1. Hyperkalemia

Hyperkalemia occurs in KTRs with an incidence of 25–44% and is usually asymptomatic [103], but it can also predispose to the onset of serious arrhythmias and sudden death, thereby influencing mortality and morbidity in these patients [104,105]. High potassium levels may be related to insulin deficiency, metabolic acidosis, and low eGFR. In KTRs, though, they are associated with renal tubular acidosis regardless of the above factors that may or may not coexist [103].

The drugs used in KTRs play a predominant role in the development of hyperkalemia. Indeed, trimethoprim/sulfamethoxazole (TMP/SMX), used in the prophylaxis of pneumocystis carinii infections (PCPs) and urinary tract infections, contributes to hyperkalemia [106]. However, low-dose prophylactic regimens with thrice-weekly administration have been associated with lower incidence rates [107].

The use of RAAS inhibitors is also associated with an increased risk of hyperkalemia [108,109,110]. In a study of 2684 kidney transplant recipients, Shin JI et al. [111] found a markedly increased risk of life-threatening hyperkalemia (>2-fold) in patients taking RAAS inhibitors. At the same time, data on patient and graft survival benefits continue to support their clinical use [103].

Among all drugs, CNIs remain the main cause of hyperkalemia in transplant recipients [103,112], determining aldosterone resistance and functional hypoaldosteronism [113]. Furthermore, experimental models have described the hyperactivation of the NCC, expressed on the distal convoluted tubule, induced by tacrolimus but not by cyclosporine [108,114,115]. This would explain the higher incidence of hyperkalemia in patients treated with tacrolimus [115]. Further studies are needed to validate this thesis; however, if this were the case, as NCC is sensitive to thiazide diuretics, these drugs would become a valid therapeutic option for hyperkalemia KTR, improving outcomes [103].

Although controlling potassium levels in the transplanted patient could represent a great benefit in terms of survival, the drugs mentioned above are often essential in post-transplant management, and their suspension or dosage adjustment is not always possible. While Belatacept and mTORis represent, when feasible, alternative options to CNIs in the case of hyperkalemia, TMP/SMX remains the first-line drug for the prevention of PCP, although alternative agents (dapsone and atovaquone) have been studied [116].

##### Treatment of Hyperkalemia

The National Kidney Foundation suggests a limited dietary intake of potassium (1–3 g/day) as a first and economical approach for patients with hyperkalemia [117].

In addition, some drugs have proven useful in counteracting the increase in potassium levels. These include diuretics and fludrocortisone, which stimulates potassium excretion in the distal nephron due to its mineralocorticoid properties [108,118]. The use of cation exchange resins, which are widely used in CKD, requires further investigation. For example, sodium polystyrene sulfonate has been associated with colon perforation, so caution is needed when using it, especially during the post-operative period [103].

Novel potassium binders such as Patiromer and Sodium Zirconium Cyclosilicate are effective in the treatment of chronic or recurrent hyperkalemia and associated with fewer gastrointestinal side effects [119,120]. Their use in transplant recipients is currently under study, also due to potential interactions with immunosuppressive drugs [121]. In fact, the use of Patiromer has been associated with an increase in serum concentrations of tacrolimus but not of ciclosporin; this effect does not occur with Sodium Zirconium Cyclosilicate, which correlates with a higher sodium load [120].

#### 2.5.2. Metabolic Acidosis

Metabolic acidosis after kidney transplantation occurs with a prevalence of 12–58% and then decreases to 13–16% after 12 months [103,122,123]. Although it can also occur in patients with normal renal function, the prevalence and persistence are higher for eGFR values < 30 mL/min/1.73 m^2^ [122]. It commonly manifests as renal tubular acidosis (RTA); the predominant forms are distal (classical) type I and distal (hyperkalemic) type IV. However, proximal renal tubular acidosis type II can also be found early after kidney transplantation due to tubular damage and hyperparathyroidism [124]. With resolution of tubular damage and hyperparathyroidism, RTA may completely decline within the first 6 months after transplantation [125].

In addition to factors traditionally implicated in the development of metabolic acidosis, mechanisms specifically associated with transplantation come into play, including reduced allograft function, donor and donation characteristics (transplant from deceased donors and longer cold ischemia times are more associated with the development of metabolic acidosis), immunological damage from rejection, hyperparathyroidism, and use of calcineurin inhibitors [103,123,124].

The latter have a dose- and duration-dependent toxicity on the renal tubule, which can therefore be modulated by reducing the dose of the drug [114]. Furthermore, as with hyperkalemia, RTA is also more associated with tacrolimus-based immunosuppression [108]. Other drugs such as mycophenolate or some antibiotics may also contribute to the development of non-anion gap metabolic acidosis due to onset of chronic diarrhea [125].

Clinically, patients with metabolic acidosis are often asymptomatic [103], but it is reasonable to assume that the consequences of metabolic acidosis in transplant recipients may be similar to those in a common subject, although data on this matter are limited [126]. Some data support the association between persistent metabolic acidosis at three months post-transplant and an increased risk of graft failure, especially for bicarbonate levels below 22 mEq/L [103,122,126].

The correlation between metabolic acidosis and anemia is also proven in transplant patients [126]. RTA is also among the possible causes of mineral and bone alterations typical of post-transplantation [103]; it has been identified as a causal factor in sarcopenia after kidney transplantation by inhibiting muscle protein synthesis, stimulating protein catabolism inversely proportional to serum bicarbonate levels, and increasing phosphaturia, thus contributing to hypophosphatemia and causing the depletion of intramuscular ATP reserves [126,127,128].

Finally, metabolic acidosis was evaluated as a risk factor for cardiovascular morbidity and mortality [129]. Presumably, the alteration of myocardial contractility and the predisposition to the development of arrhythmias observed in the context of acidosis may explain this association [126].

##### Treatment of Metabolic Acidosis

Although it is clear that metabolic acidosis can negatively impact both patient and allograft survival, there are conflicting opinions on the possible treatment. Some have even associated sodium bicarbonate therapy with a higher risk of graft failure [130]; for others, this supplement is safe and not associated with any particular side effects [131]. Great expectations were poured into a large multicenter randomized controlled trial (Preserve-Transplant) conducted between 2017 and 2019 with the aim of verifying whether the correction of acidosis helps to preserve the functionality of the renal transplant and decrease the progression of renal disease in the transplant recipient. However, at the end of the 2 years of observation, it was concluded that the correction of metabolic acidosis by treatment with sodium bicarbonate does not influence the decline in renal function; therefore, it should not be recommended for this purpose [132].

Equally limited are the data on the benefits of targeted dietary changes. A diet rich in fruit and vegetables would correlate with lower all-cause mortality, just as a lower intake of animal protein could be an effective and economical method to improve acidosis in the post-transplant period [103,133].

#### 2.5.3. Hypomagnesemia

In kidney transplant recipients, hypomagnesemia occurs with high prevalence. In most cases, magnesium levels reach their lowest value within the second month after transplantation and then progressively increase. However, in 20% of patients, hypomagnesemia may persist for many years [103,134].

As with other metabolic disorders described so far, CNIs play a fundamental role, non-dose-dependent [103], and the incidence of hypomagnesemia is higher among patients treated with tacrolimus than with ciclosporin [103,135]. mTOR inhibitors can also cause hypomagnesemia, although less significantly than CNIs [136]. The specific mechanisms by which this occurs have not been well defined due to conflicting results [136,137] Many other drugs used in the post-transplant period may contribute to the development of hypomagnesemia. These include steroids, diuretics, proton pump inhibitors, as well as several drugs approved for the treatment of post-transplant bone mineral disease (teriparatide, bisphosphonates, and denosumab) [103,136].

Hypomagnesemia can manifest itself with more or less serious symptoms, from tremors to tetanic and convulsive crises, and from isolated electrocardiographic alterations to ventricular arrhythmias in patients with pre-existing heart disease [103]. Furthermore, some data highlights the correlation between hypomagnesemia, vascular stiffness, and reduced graft survival, as well as between hypomagnesemia and insulin resistance [103,134,138]. This last aspect is particularly relevant in light of the increasing incidence of post-transplant diabetes. Huang et al. [139] demonstrated that plasma magnesium values < 1.8 mg/dL were associated with an increased risk of post-transplant diabetes, therefore involving an increased risk of cardiovascular complications and mortality, resulting in poorer transplant patient outcomes [140].

##### Treatment of Hypomagnesemia

There are conflicting opinions on the treatment of hypomagnesemia with magnesium supplementation. In some studies, serum magnesium levels normalized after supplementation; in others, no significant improvement was observed [103,141,142]. Furthermore, while magnesium supplementation has led to an improvement in glucose metabolism and insulin sensitivity in diabetic patients, the same results have not been reported in kidney transplant recipients [103].

It would be advisable to orient patients with mild hypomagnesemia, without risk factors, towards a higher dietary intake of magnesium (broad-leaf vegetables, legumes, nuts, and whole grains) [103,143]. In cases of moderate (<1.5 mg/dL) or severe (<1.2 mg/dL) hypomagnesemia, with or without risk factors (e.g., pre-existing heart disease), magnesium supplementation is recommended, first intravenously and then orally [103,143].

### 2.6. Mineral Bone Disease

After renal transplantation, bone and mineral metabolism disorders are common and represent important causes of morbidity and mortality [144,145], as well as determining factors for graft survival [146]. In the post-transplant period, bone resistance is significantly compromised due to alterations affecting the bone in the short- and long term [144]. These are identified in post-transplant bone and mineral disease (PTBD), a generic term used to indicate multiple bone changes, including osteopenia (32%), osteoporosis (15%), renal osteodystrophy, osteonecrosis, and fractures [144,147,148]. In the first 6 and 12 months after kidney transplantation, a significant decrease in bone mineral density (BMD) occurs in the peripheral but not in the central skeleton, with involvement of both cortical and trabecular bone [147,149]. In the following 12–18 months, drastic decreases in bone density are detected, mainly in the spine and hip [144,150]. In the long term, there is a stabilization of BMD, which would progressively increase by approximately 6% between the sixth and tenth years after the transplant and to a lesser extent (2%) in the following years [144].

All this translates into a significantly increased risk of fracture, especially in the first 3 years, with a significant impact on the morbidity and mortality of recipients [145,151,152].

The most frequently encountered fractures are those of the spine, hip, and extremities (hand, radius/ulna, or foot/ankle) [144]. The risk of mortality is higher for vertebral fractures, while hip and extremity fractures are more closely related to the risk of graft loss [152].

The pathophysiology of PTBD, as for pre-transplant bone and mineral disease, is based on alterations in parathyroid hormone (PTH), vitamin D, and calcium–phosphorus balance [153,154,155]. Added to all this are aggravating factors related to the transplant, including the immunosuppressive regimens adopted [147].

#### 2.6.1. Hyperparathyroidism and Vitamin D Deficiency

A successful kidney transplant should correct the abnormalities responsible for secondary hyperparathyroidism to CKD [103,156]. However, parathyroid involution does not always occur immediately [103]. According to some studies, in most cases, PTH levels after transplantation decrease in the first 3 months, slowly increase within a year, and then decrease again [157,158].

Pre-transplant PTH levels significantly influence the persistence of hyperparathyroidism [159]. In fact, in patients with severe pre-transplant hyperparathyroidism (often due to the presence of adenomas or parathyroid hyperplasia), PTH values remain elevated for longer [103,157]. Another important factor is the degree of the allograft functionality, which in turn influences the restoration of vitamin D levels [144,160]. Vitamin D deficiency occurs in 80% of recipients within 3 months of transplantation [161], causes hypocalcemia, and activates the cascade of events culminating in PTH release and bone remodeling [162]. All this explains why complete resolution of hyperparathyroidism does not occur in all cases but only in 30% of cases within the first year and in 57% of cases within the second year after transplantation [144].

This finding is of particular importance since persistent hyperparathyroidism has been associated with a high risk of developing bone and mineral disease, as well as an increased incidence of fractures [147].

#### 2.6.2. Hypercalcemia and Hypophosphatemia

Hypercalcemia and hypophosphatemia are important biochemical abnormalities that occur after transplantation [163]. In transplant recipients, the persistence of a PTH above the range, associated with a recovery of renal function, even if not complete in the first few months, represents a predisposing factor for hypercalcemia. The occurrence of hypercalcemia after renal transplantation exhibits substantial variability and may reach significant levels [103]. About 59% of transplant recipients are hypercalcemic at 3 months; in 45% and 21% of recipients, respectively, hypercalcemia persists at 1 and 5 years [144]. After transplant, calcium levels decrease in the first 7 days and then increase significantly within a month [147], reaching maximum values in the first 2 months after the transplant and remaining high in 18% of cases for at least 12 months and in 6% of cases up to 10 years [164]. This occurs regardless of pre-transplant PTH levels [147]. Pre-existing hyperparathyroidism, therefore, can be considered a protective factor for hypocalcemia in the first week and a risk factor for hypercalcemia after the first week [165].

Hypercalcemia in transplant recipients is also related to other factors [103,144]. These include the improvement of post-transplant calcitriol production and the duration of pre-transplant dialysis due to the reabsorption of calcium phosphate deposits in the soft tissues that form in patients undergoing dialysis [15,103].

Since post-transplant hypercalcemia is mild–moderate and chronic in nature, the symptoms associated with it are often not evident [103]. However, hypercalcemia can be associated with the development of calcifications in the allograft that influence the survival of the graft [144,166].

Along with alterations in serum calcium, changes in phosphatemia are characteristic and relevant in the post-transplant period. Phosphorus levels typically decrease in the first 3 months after transplant and then gradually increase over 3 to 12 months [144,147]. The data indicates that the incidence of hypophosphatemia varies from 40 to 93%, with maximum values in the second week after transplantation and a tendency to regression within 1 year [103,144,161]. Phosphate homeostasis is regulated by dietary phosphate, PTH, vitamin D, insulin, and fibroblast growth factor 23 (FGF-23) [103]. Specifically, FGF-23 levels are also elevated in patients with chronic kidney disease and may take time to normalize after successful kidney transplantation [103]. In most cases, the FGF-23 level decreases in the first 3 months after transplantation and remains stable thereafter [147].

There is a positive correlation between higher FGF-23, higher PTH, and lower serum phosphate level at 3 months after transplantation [147]. In fact, elevated PTH can determine elevated FGF-23 and indirectly an increase in phosphaturia and accelerated bone demineralization [147]. However, other factors also contribute to hypophosphatemia: tacrolimus (acting on the phosphate cotransporter NaPi-2a in the renal tubule), the duration of dialysis, low levels of calcitriol, high levels of PTH, and tubular damage from immunosuppression [103]. The clinical consequences of hypophosphatemia, especially in severe forms, are worrying for the transplant population [103]; at the same time, no association was found between phosphate loss and adverse graft outcomes [167]. Conversely, recent studies show favorable outcomes in patients with hypophosphatemia, indicating a very well-functioning graft [168].

#### 2.6.3. Immunosuppressive Regimens and Development of Post-Transplant Bone Mineral Disease

In recent decades, the epidemiology of post-transplant bone mineral disease has been modified by changes in immunosuppressive regimens [144]. A cohort study conducted between 1994 and 2009 to estimate the 3-, 5-, and 10-year cumulative incidence of nonvertebral fractures (proximal humerus, forearm, and hip) in adult kidney transplant recipients found lower fracture rates over the past decade [151]. Certainly, the minimization or avoidance of glucocorticoids in immunosuppressive regimens, being associated with a smaller reduction in BMD, influences the reduction in the cumulative incidence of fractures of all types [151,169].

Monier-Faugere et al. have shown that cumulative doses of prednisolone are negatively correlated with bone volume and bone turnover, unlike cumulative doses of cyclosporine or azathioprine [170]. These data have been confirmed by studies comparing standard immunosuppressive regimens with those with early glucocorticoid withdrawal [144].

A USRDS analysis of 77,430 transplant patients showed that patients treated with immunosuppressive regimens with early steroid withdrawal had a fracture risk < 26% at 1 year after transplant, a fracture risk < 70% at 3 years after transplantation, and a lower risk of fractures associated with hospitalization [169]. Specifically, the qualitative bone alterations most typically identified are low trabecular bone volume, low bone turnover, and mineralization defects [170,171]. Regarding bone turnover, the transplant itself is associated with a reduction in it [144]. All this would explain why, in the most recent studies, in which the transplanted population analyzed is subjected to the new therapeutic regimens of rapid suspension and/or minimization of steroid, low bone turnover remains a common finding, while bone volume is less seriously compromised [144].

#### 2.6.4. Diagnosis of Post-Transplant Bone Mineral Disease

The KDIGO 2017 guidelines recommended assessing BMD in kidney transplant recipients using dual-energy X-ray absorptiometry (DXA) to predict fracture risk [172]. DXA should be performed 2–4 months after transplant, annually until BMD is stabilized, and every 3–5 years thereafter [144].

Further studies have shown that BMD measurement, combined with an assessment of bone turnover, would have discriminatory capacity in predicting the risk of fracture in transplant recipients [173,174]. Circulating markers of bone formation and resorption are PTH and bone-specific alkaline phosphatase (BSAP) [144]. A PTH < 2 times the upper limit of normal and a BSAP less than the lower limit of the reference range are indicative of low bone turnover. In the case of discordant PTH and BSAP, bone biopsy is recommended to guide therapy [144].

Biopsy including double labeling with tetracycline is the gold standard for the diagnosis of post-transplant bone mineral disease and more specifically of the type of characteristic bone alteration [148].

The KDIGO 2017 guidelines recommended bone biopsy as a guide to the most appropriate treatment for all patients with eGFR > 30 mL/min within 12 months of transplantation [172]. However, this is an invasive test and is not always feasible. The use of bone turnover markers has proven to be a practical alternative, albeit with lower specificity. Of the two most widely used markers, BSAP has proven to be more reliable than PTH in reflecting the underlying bone turnover [147].

#### 2.6.5. Therapeutic Management of Post-Transplant Bone Mineral Disease

The treatment of biochemical alterations related to PTBD is essential to prevent its development, therefore reducing the risk of fracture and the rate of mortality and morbidity [144].

Around 80% of transplant recipients have hypovitaminosis D; therefore, the KDIGO recommendations include starting therapy with calcitriol, in the first year of transplant, for eGFR > 30 mL/min/1.73 m^2^ and low BMD [172]. This is effective in reducing PTH levels, although hypercalcemia and hypercalciuria are relatively common side effects [175,176]. Additionally, vitamin D supplementation was associated with increased BMD at the spine and femoral neck in the first year after transplantation [176]. There are no studies aimed at evaluating the effects of vitamin D on BMD and fracture risk after 1 year [144].

In the case of persistent hyperparathyroidism, the use of cinacalcet has proven effective [177]. At the same time, no studies have shown the efficacy of cinacalcet in reducing the risk of fracture in kidney transplant recipients. Therefore, although cinacalcet is useful in controlling biochemical abnormalities associated with bone mineral disease in CKD, it is not approved by the U.S. Food and Drug Administration for the treatment of PTBD [144].

Compared to cinacalcet, parathyroidectomy has been shown to be more effective in controlling PTH and calcium levels, also showing beneficial skeletal effects, such as an increase in BMD and a reduction in bone resorption markers [178].

Bisphosphonates are certainly the most studied drugs for PTBD treatment. They increase BMD by suppressing bone turnover through osteoclast inhibition [144]. In particular, an improvement in BMD has been shown at the femoral neck and lumbar spine, especially for treatments started in the first 6 months after transplantation [179]. However, their use remains controversial for various reasons: there are conflicting data on the risk of inducing adynamic bone disease [180,181]; no study has evaluated the effects of bisphosphonates on BMD beyond 12 months after transplantation [144]; cases of acute renal failure secondary to the use of intravenous bisphosphonates have been reported [182]; they can cause adverse events such as osteonecrosis of the jaw, atypical femoral fracture, and severe hypocalcemia [147]; their administration should be carefully evaluated in relation to renal function; and they are generally avoided in patients with eGFR < 30 mL/min/1.73 m^2^ [172].

The latest frontier in the treatment of PTBD is represented by the use of antiresorptive and osteoanabolic agents. Two drugs are currently being studied: denosumab and teriparatide.

The effects of denosumab have been evaluated in a prospective randomized study in kidney transplant recipients that showed a significant increase in BMD at the lumbar spine and hip after 1 year of treatment [183]. These data were confirmed by the FREEDOM study, which also found a reduction in the risk of vertebral, non-vertebral, and hip fractures [184]. Urinary tract infections and hypocalcemia have been reported among the most relevant adverse events associated with denosumab [144]. Importantly, denosumab can be used irrespective of eGFR, but baseline serum calcium, vitamin D, and magnesium must be optimized before administration to minimize the risk of hypocalcemia [172]. Further studies are needed to evaluate the effect of denosumab beyond 1 year of transplantation.

It is reasonable to consider the use of denosumab as well as bisphosphonates when there is clinical and biochemical evidence of high-turnover PTBD, with the aim of maintaining bone quality and quantity while reducing the risk of fracture [147]. However, given the risks associated with the use of these drugs, treatment should be limited to cases with a certain diagnosis (through bone biopsy).

There are conflicting data regarding the efficacy of teriparatide. In a randomized double-blind controlled study, no significant benefit in terms of BMD or superiority over alternative therapies emerged after kidney transplantation [185]. Teriparatide is generally considered for patients with low-turnover bone disease, but its use in advanced CKD (eGFR < 30 mL/min/1.73 m^2^) is not routinely recommended due to limited evidence and increased risk of hypercalcemia and other adverse effects in the context of reduced renal function [172]. Therefore, teriparatide could be considered in the case of low bone turnover to prevent further loss of BMD, but we must consider that adverse effects such as hypercalcemia and hyperuricemia are dose-dependent and of an intensity that is inversely proportional to eGFR values [186].

Finally, given the skeletal effects of glucocorticoids, it would be advisable, when possible, to reduce the doses below the skeletal toxicity threshold (<7.5 mg per day), especially in patients considered to be at high risk of fracture (female gender, diabetics, and age > 55 years) [144,187].

## 3. Summary and Conclusions

Table 1 synthesizes the non-immunological risk factors in KT.

As highlighted throughout this extensive review, the most prevalent risk factors associated with native CKD also play a significant role in KT patients. This evidence was also directly provided in an observational propensity-score-based analysis comparing the prognostic role of traditional CKD risk factors on kidney failure in a cohort of 757 KT patients versus 1940 native CKD patients matched for age, gender, eGFR, and proteinuria at baseline [188]. In that study, the KT patients were enrolled 12 months after surgery to limit the effect modification of immunological risk factors on traditional CKD risk factors. The analysis showed that, although the percentage of KF events explained by the non-immunological risk factors was higher in native CKD (70%) than KT (31.2%), the main predictors of KF (namely eGFR, proteinuria, history of CVD, and use of RAAS inhibitors) remained significantly associated with the primary outcome in KT patients. Moreover, dialysis vintage (i.e., the duration of dialysis before KT) added further risk to KF development on top of the traditional risk factors. Moreover, other non-immunological complications related to long-term immunosuppression in KT patients should not be overlooked. The condition of kidney transplant recipient merits extensive and multidisciplinary management considering the balance between the risk of rejection and risk of immunosuppression: the long-term follow-up of this category of patients includes surveillance of malignancies (renal masses and solid tumors, lymphoproliferative disorders, and skin cancers) [189,190], chronic allograft nephropathy, opportunistic infections, cardiovascular complications, and neurotoxicity. These factors are dynamically interrelated over time and may affect the overall prognosis, graft survival, and therapeutic choices beyond nephrological management alone.

In conclusion, KT patients represent a complex category, requiring multidisciplinary management of the main traditional risk factors for CKD, immunological risk of rejection, and complications related to immunosuppression. For this reason, the inclusion of KT patients in large randomized clinical trials in CKD is warranted to validate specific findings in this population and guide clinical practice. The current randomized studies in CKD, in fact, explore kidney and CV outcomes concomitantly. This means that patients at high CV risk may benefit from further study and new drugs. Given their history of past KF and the residual CV and kidney risk due to the reported comorbidities, KT patients clearly belong to this category of high-risk patients.

## Figures and Tables

**Table 1:** Non-immunological risk factors in KT. GFR, glomerular filtration rate; CKD, chronic kidney disease; RAAS, renin–angiotensin–aldosterone system; ESA, erythropoiesis-stimulating agent; HIF-PH, hypoxia-inducible factor prolyl hydroxylase; CCBs, calcium channel blockers; RAASi, renin–angiotensin–aldosterone system inhibitor; SGLT2i, sodium–glucose cotransporter-2 inhibitor; nsMRAs, nonsteroidal mineralocorticoid receptor antagonists; CNI, calcineurin inhibitor; MACE, major adverse cardiovascular event; GLP-1RA, glucagon-like peptide-1 receptor agonist; DPP-4i, dipeptidyl peptidase-4 inhibitor; mTORi, mammalian target of rapamycin inhibitor; RTA, renal tubular acidosis; SHPT, secondary hyperparathyroidism; FGF-23, fibroblast growth factor-23; PPI, proton pump inhibitor; TMP/SMX, trimethoprim–sulfamethoxazole; ECG, electrocardiogram.

Risk Factor	Clinical Impact	Mechanism	Therapeutic Management
Anemia	• Reduced GFR • Graft failure • Cardiovascular morbidity and mortality	• Chronic graft hypoxia leading to fibrosis and inflammation • Chronic inflammatory status (IL-6 and TNF-α activation) • Increase in the cardiac output leading to left ventricular eccentric hypertrophy and arterial remodeling	• Iron supplementation • ESAs • HIF-PH inhibitors
Hypertension and sodium intake	• Graft failure • Cardiovascular morbidity and mortality	• Traditional risk factors (age, male gender, augmented BMI, insulin resistance, obstructive sleep apnea, smoking habit) • CKD-related factors (upregulation of RAAS, increased SNA activity, endothelial dysfunction, arterial stiffness, ESA therapy) • Donor related factors (age, pre-existing hypertension, obesity, poor graft quality, gene polymorphisms) • Immunosuppressive therapy (hydro-saline retention, increased vascular resistance, arrhythmias, atherosclerosis, high doses of glucocorticoids, direct vascular effects of calcineurin inhibitors) • Transplant renal artery stenosis • Antibody-Mediated Rejection • Primary hyperaldosteronism	• Na^+^ restriction • CCBs • RAASi • Beta blockers • Diuretics • SGLT2i • nsMRAs
Diabetes	• Post-transplant infections • Delayed graft function • Infections • Early onset of rejection • Diabetic kidneys disease • Increased MACE risk	• CNI/steroid-induced insulin resistance and β-cell damage • Infections (hepatitis C) • Hypomagnesemia • Resolution of uremic anorexia • Increased body weight	• Lifestyle control • Metformin • Sulfonylureas and glinides • GLP-1RA • DPP-4i • SGLT2i • Insulin
Proteinuria	• Independent risk factor for reduced graft survival, GFR decline and patient mortality	• Donor related factors (female sex, advanced age, cardiovascular death, proteinuria) • Recipient related factors (male sex, acute rejection, chronic rejection) • Graft glomerulopathy • mTORi toxicity	• mTORi discontinuation • RAASi • Dietary protein restriction
Electrolyte and acid–base disturbances	• Hyperkalemia: arrhythmias and sudden death • Hypomagnesemia: tremors, tetanic/convulsive crises, isolated ECG alterations, ventricular arrhythmias • Metabolic acidosis: risk of graft failure, anemia, cardiovascular morbidity and mortality, arrhythmias	• Hyperkalemia: related to insulin deficiency, metabolic acidosis, low eGFR, renal tubular acidosis, TMP/SMX therapy, RAASi, NCC hyperactivation induced by tacrolimus • Hypomagnesemia: related to CNI/mTORi therapy, steroids, diuretics, PPI, teriparatide, bisphosphonates and denosumab • Distal/proximal RTA, hyperparathyroidism, reduced allograft function, longer cold ischemia times, immunological damage from rejection, CNI, mycophenolate, antibiotics	• K^+^ dietary restriction (1–3 g/day);diuretics/fludrocortisone; new K^+^ binders • Higher dietary magnesium intake, magnesium supplementation (IV/orally) • Treat causes; bicarbonate as needed; dietary alkali.
Mineral bone disease	• Hyperparathyroidism and vitamin D deficiency • Hypercalcemia and hypophosphatemia	• Persistent SHPT, low Vitamin D, high FGF-23 • Hypercalcemia: SHPT persistence associated with recovery of renal function, improvement of calcitriol production, reabsorption of calcium phosphate deposits in the soft tissues • Hypophosphatemia: SHPT and FGF-23 excess determine phosphaturia, tacrolimus, low calcitriol, tubular damage from immunosuppression	• Vitamin D/calcitriol • Cinacalcet or surgery • Bisphosphonates • Consider antiresorptives (Denosumab, Teriparatide) • Minimize steroids

## Data Availability

No new data were created or analyzed in this study.

## Abbreviations

The following abbreviations are used in this manuscript:

ABMR	Antibody-Mediated Rejection
ACE-i	Angiotensin-Converting Enzyme Inhibitor
AKI	Acute Kidney Injury
ANZDATA	Australia and New Zealand Dialysis and Transplant Registry
ARBs	Angiotensin Receptor Blockers
ATIIR	Angiotensin II Type 1-Receptor Activating
BMD	Bone Mineral Density
BMI	Body Mass Index
BSAP	Bone-Specific Alkaline Phosphatase
BP	Blood Pressure
CCBs	Calcium Channel Blockers
CGM	Continuous Glucose Monitoring
CKAD	Chronic Kidney Allograft Disease
CKD	Chronic Kidney Disease
CNIs	Calcineurin Inhibitors
CT	Computed Tomography
CV	Cardiovascular
CVD	Cardiovascular Disease
CsA	Cyclosporine A
DCGL	Death-Censored Graft Loss
DGF	Delayed Graft Function
DM	Diabetes Mellitus
DPP-4	Dipeptidyl Peptidase-4
DXA	Dual-Energy X-Ray Absorptiometry
ECF	Extra-Cellular Fluid
eGFR	Estimated Glomerular Filtration Rate
EPO	Erythropoietin
ESA	Erythropoiesis Stimulating Agent
ESKD	End-Stage Kidney Disease
FGF-23	Fibroblast Growth Factor-23
GF	Graft Failure
GIP	Glucose-Dependent Insulinotropic Polypeptide
GLP1-RAs	Glucagone-Like Peptide 1-Receptor Agonists
Hb	Hemoglobin
HIF-PH	Hypoxia-Inducible Factor-Prolyl Hydroxylase
HT	Hypertension
IV	Intravenous
KDIGO	Kidney Disease Improving Global Outcomes
KF	Kidney Failure
KTRs	Kidney Transplant Recipients
KT	Kidney Transplantation
LVH	Left Ventricular Hypertrophy
MACE	Major Adverse Cardiovascular Event
MBD	Mineral Bone Disease
MMF	Mycophenolate Mofetil
mTORi	Mammalian Target of Rapamycin Inhibitor
NCC	Sodium Chloride Cotransporter
nsMRA	Nonsteroidal Mineralcorticoid Receptor Antagonist
PCP	Pneumocystis Carinii Infection
PDTM	Post-Transplant Diabetes Mellitus
PTH	Parathiroid Hormone
PTA	Post-Transplantation Anemia
PTBD	Post-Transplant Bone and Mineral Disease
PTRA	Percutaneous Transluminal Balloon Renal Angioplasty
RAAS	Renin–Angiotensin–Aldosterone System
RCT	Randomized Controlled Trial
RTA	Renal Tubular Acidosis
SGLT-2i	Sodium Glucose Cotransporter-2 Inhibitor
SNA	Sympathetic Nervous Activity
SRL	Sirolimus
TLA	Three-Letter Acronym
TMP/SMX	Tripethoprim/Sulfamethoxazole
TRAS	Transplant Renal Artery Stenosis
WHO	World Health Organization
WNK	With-No-Lysine Kinase
